# DeepGuide: a novel wavelength-specific navigation system for membrane visualization in surgery

**DOI:** 10.1097/JS9.0000000000002370

**Published:** 2025-04-01

**Authors:** Zehong Chen, Xuejie Li, Zongheng Zheng, Jianpei Liu, Jiafeng Fang, Jianglong Huang, Tufeng Chen, Hongbo Wei

**Affiliations:** Department of Gastrointestinal Surgery, The Third Affiliated Hospital of Sun Yat-sen University, Guangzhou, China

**Keywords:** membrane anatomy, single-band light source, intraoperative navigation

## Abstract

**Background:**

Membrane anatomy (MA) has become pivotal in modern surgery, enabling surgeons to operate within avascular natural planes to minimize concomitant injury. However, intraoperative identification of membranous structures remains challenging. Poor membrane visualization compromises both radical resection and functional preservation, underscoring the need for innovative solutions.

**Methods:**

In this study, we develop a innovative tool, DeepGuide, to help surgeon make membrane visualization during surgery. Moreover, we design a single-arm prospective study to validated its effectiveness. A total of 60 patients undergoing laparoscopic radical resection for gastrointestinal tumors at The Third Affiliated Hospital of Sun Yat-sen University were enrolled. Primary outcomes included signal-to-background ratio (SBR), mesenteric integrity, absorbance of membrane specimens, and immunofluorescence staining of collagen components.

**Results:**

DeepGuide significantly improved mesenteric integrity rates from 75% (conventional laparoscopy) to 98%. SBR under DeepGuide (2.30 ± 0.15) surpassed conventional lighting (1.32 ± 0.16). Absorbance analysis revealed reduced light absorption at 385 nm. Immunofluorescence confirmed collagen I/III enrichment in mesenteric submucosa, aligning with 385 nm-excited autofluorescence properties.

**Conclusion:**

DeepGuide revolutionizes intraoperative membrane visualization without requiring contrast agents. It enhances surgical precision and supports MA principles. These findings highlight DeepGuide’s potential to redefine standards in oncologic surgery.

## Background

Surgical procedures have entered the era of membrane anatomy (MA), where operating within avascular natural spaces guided by MA principles has become a hallmark of surgical excellence. The seminal work of Professor Richard J. Heald exemplifies this paradigm through total mesorectal excision (TME), where adherence to the “holy plane” and recognition of “angel hair” ensure mesorectal integrity for optimal oncological and functional outcomes^[^1^]^. While MA-driven approaches have revolutionized multiple specialties including gastroenterology and gynecology^[^2-4^]^, critical limitations persist. Clinical evidence reveals that current MA-based procedures achieve only 75% mesenteric integrity in colorectal cancer TME/CME cases^[^5,6^]^, primarily due to intraoperative difficulties in precise membrane identification. This technological gap severely compromises both therapeutic efficacy and functional preservation. To address this fundamental challenge, we developed DeepGuide – an innovative wavelength-specific membrane recognition and navigation system.

**HIGHLIGHTS**

- **Question**: Can a wavelength-specific navigation system improve intraoperative membrane visualization and surgical outcomes?
- **Findings**: In this study of 60 patients, DeepGuide achieved 98% mesenteric integrity (vs. 75% with conventional laparoscopy) and significantly higher optical contrast. Collagen autofluorescence at 385 nm underpinned its efficacy.
- **Meaning**: DeepGuide offers a practical, agent-free solution for membrane visualization, advancing precision in MA-guided surgery.

## Methods

### Study design

The DeepGuide navigation system integrates a 385 nm (A wavelength of 385 nm with a full width at half maximum [FWHM] of 10 nm) single-band light source module into the traditional laparoscopic system. According to previous literature, the mesenteric integrity rate in conventional laparoscopic surgery remains only 75% (as per the Dutch Colorectal Cancer Group standard)^[^7^]^. We anticipate DeepGuide will elevate that ratio to 90%, setting the study’s alpha level to 0.05, maintaining beta confidence at 0.2, and accounting for an estimated 10% patient dropout rate. Based on these parameters, a total of 60 cases were calculated to be required for this single-arm study. Therefore, we ultimately enrolled 60 gastrointestinal tumor patients undergoing laparoscopic radical surgery at the Third Affiliated Hospital of Sun Yat-sen University between September 2024 and January 2025. Detailed inclusion/exclusion criteria are documented in the Clinical Trial Registry (ChiCTR2400089718).

### Research outcomes

The research outcomes comprised: (1) signal-to-background ratio (SBR, defined as the grayscale value ratio between the mesentery-mesenteric bed interface and adjacent mesenteric areas, calculated by ImageJ software); (2) integrity rates of the mesentery and mesenteric bed (assessed per Nagtegaal scoring^[^7^]^, categorized as complete, relatively complete, and incomplete); and (3) mesenteric absorbance(measured by UV-visible spectrophotometer). (4) immunofluorescence staining of collagen components in mesentery specimens. The light source switching to DeepGuide mode when separating membrane. All surgeries are performed by the project leader. The complete surgical workflow was video-recorded for postoperative review and analysis.

The SBR values were calculated and compared between DeepGuide and traditional laparoscopic illumination; statistical analysis employed paired t-tests using SPSS 26.0 (*α* = 0.05). Postoperative specimens underwent Nagtegaal scoring by two independent senior pathologists. Concurrently, mesenteric samples from 20 surgical cases were collected for absorbance measurements.

## Results

**Figure 1.**
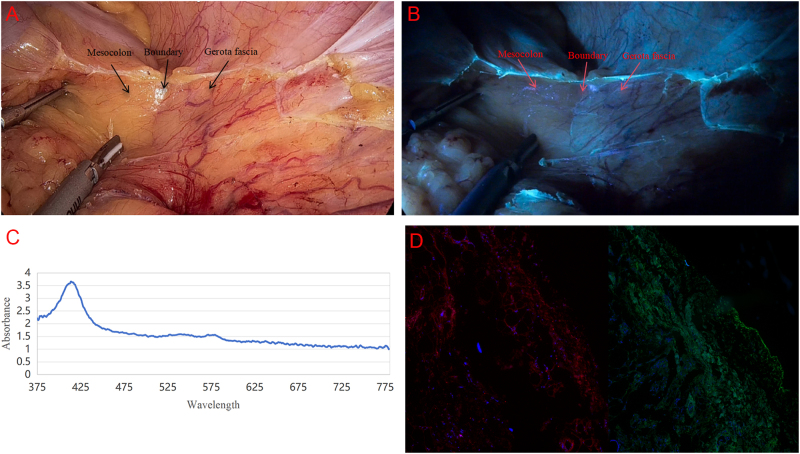
(A) Surgical images under traditional laparoscopic light source, the boundary between mesocolon and gerota fascia is hard to identified and easy to go through incorrect plane. (B) Surgical images under DeepGuide illumination, the boundary between mesocolon and gerota fascia showed obviously and helpful to correct plane. (C) The absorbance of mesocolon at different wavelengths. (D) Immunofluorescence staining of collagen structure in mesenteric specimens.

As shown in Fig. 1A, traditional laparoscopic light sources fail to clearly identify the fascia. Under such illumination conditions, the boundaries of the fascial layer appear blurred, which may lead surgeons to inadvertently enter incorrect planes and cause complications. In contrast, Fig. 1B demonstrates that with DeepGuide assistance, the fascial structure appears clear and continuous, exhibiting high contrast that effectively guides surgeons into the membrane plane while adhering to MA principles.

We further conducted quantitative analyses, with results revealing that compared to the 75% mesenteric integrity rate achieved with conventional laparoscopic lighting, DeepGuide-navigated surgeries attained a 98% mesenteric integrity rate, significantly enhancing both membrane preservation and surgical quality (Table 1). Subsequent comparisons of total SBR between DeepGuide and traditional laparoscopic systems (Table 2) showed significantly higher SBR values for DeepGuide (*P* < 0.001).

**Table 1:** Mesenteric integrity rate under two different light source conditions

Characteristic	DeepGuide	Traditional
Mesenteric integrity rate	98%	75%

**Table 2:** SBR under two different light source conditions

Characteristic	DeepGuide (M ± SD)	Traditional light source (M ± SD)	*t*	*p*
SBR	2.30 ± 0.15	1.32 ± 0.16	−40.67	<0.001

To investigate the underlying mechanism, we collected membrane samples from 20 postoperative specimens for absorbance measurements. As illustrated in Fig. 1C, mesenteric samples exhibited marked absorbance reduction near 385 nm wavelength, indicating enhanced structural reflectivity at this spectral range. This low-absorbance property enables 385 nm light to penetrate superficial tissue layers, amplifying optical contrast at the membrane-bed interface and visually delineating continuous fascial boundaries during surgery (Fig. 1B).

DeepGuide’s imaging enhancement mechanism relies not only on wavelength selection but also correlates with the membrane’s biological composition. Immunofluorescence staining specifically labeling COL-1 and COL-3 in mesenteric specimens revealed continuous dense fluorescence signals in the mesothelial sublayer (Fig. 1D), confirming collagen enrichment in this anatomical region. Supporting studies indicate that collagen’s spontaneous fluorescence peaks near 385 nm^[^8^]^. As the primary structural protein in fascial layers, collagen demonstrates 385 nm-excitable autofluorescence through hydroxypyridine and lysine pyridine groups, generating intense background signals. Simultaneously, collagen’s ordered fiber arrangement creates smooth membrane surfaces that minimize absorbance (Fig. 1C) while maximizing light reflectivity. This dual mechanism – combining spontaneous fluorescence excitation with low-absorbance reflection – synergistically enhances surgical field contrast at membrane-bed interfaces, providing real-time anatomical guidance.

## Discussion

DeepGuide technology has unique innovation and advantages. Firstly, it is completely different from early membrane recognition technologies that relied on artificial intelligence^[^9^]^. DeepGuide appears intuitive and clear, and can significantly reduce the learning curve of the model. Secondly, compared to existing tools such as near-infrared fluorescence ICG imaging^[^10^]^, DeepGuide focuses on specific wavelengths, does not require injection of additional contrast agents, has no drug side effects, and is easy to operate, making it an intuitive and simple MA guidance tool.

Our report introduces a new technology for membrane recognition during surgical procedures, which may be a revolutionary breakthrough in the field of MA. We found that 385 nm is the key wavelength for membrane recognition, which suggests that other structures such as nerves, blood vessels, and lymphatic systems may also have specific recognition wavelengths because they contain vastly different biological components. In the future, DeepGuide is expected to develop into a multi wavelength integrated system that can simultaneously recognize functions such as nerves and blood vessels. Compared with technologies that rely on machine learning or fluorescence injection, DeepGuide is more intuitive and convenient for guiding MA surgery, and is expected to revolutionize MA and even surgical technology development.

## Data Availability

The data from this study can be used upon reasonable request, please contact our research team.

## Research registration unique identifying number (UIN)

The detailed content of the study is documented in the Clinical Trial Registry (http://www.chictr.org.cn, ChiCTR2400089718).
